# A 4-Week Repeated Oral Dose Toxicity Study of Ssanghwa-Tang in Crl:CD Sprague Dawley Rats

**DOI:** 10.1155/2019/2135351

**Published:** 2019-05-16

**Authors:** Sae-Rom Yoo, Hyekyung Ha, Mee-Young Lee, Hyeun-kyoo Shin, Su-Cheol Han, Chang-Seob Seo

**Affiliations:** ^1^Herbal Medicine Research Division, Korea Institute of Oriental Medicine, 1672 Yuseongdae-ro, Yuseong-gu, Daejeon 34054, Republic of Korea; ^2^Clinical Medicine Division, Korea Institute of Oriental Medicine, 1672 Yuseongdae-ro, Yuseong-gu, Daejeon 34054, Republic of Korea; ^3^Jeonbuk Department of Inhalation Research, Korea Institute of Toxicology, 30 Baekhak 1-gil, Jeongeup-si, Jeollabuk-do 56212, Republic of Korea

## Abstract

Ssanghwa-tang (SHT), a traditional herbal formula, has been widely used to recover fatigue or consumptive disease after an illness. Along with much attention to herbal formula, the concerns about the safety and toxicity have arisen. To establish the safety information, SHT was administrated in Crl:CD Sprague Dawley rats at a daily dose of 0, 1000, 2000, and 5000 mg/kg for 4 weeks. During the test periods, we examined the mortality, clinical observation, body weight change, food consumption, organ weights, hematology, serum biochemistry, and urinalysis parameters. No changes of mortality and necropsy findings occurred in any of the groups during the experimental period. In either sex of rats treated with SHT at 5000 mg/kg/day, changes were observed in food intake, reticulocyte, total bilirubin, some urinalysis parameters, and relative organ weights. The results indicated that SHT did not induce toxic effects at a dose level up to 2000 mg/kg in rats. This dosage was considered no observed adverse effect level (NOAEL) and was appropriate for a 13-week subchronic toxicity study.

## 1. Introduction

Along with the increased use of herbal medicine, safety has been highlighted. Although herbal medicine has been assumed to be safe, most of the drugs may cause a side effect. To ensure the safe use of herbal medicine, there is a need to establish a safe dose range, target organ toxicity, and therapeutic window.

Ssanghwa-tang (SHT, shuanghe-tang in Chinese, Souwa-to in Japanese), a traditional herbal formula, has been widely used in Korea for overcoming fatigue or consumptive disease after an illness [1]. In recent studies, SHT had shown several pharmacological effects including anti-inflammatory [2], antiosteoporotic [3], and antimelanogenic activities [4].

Toxicity tests are essential for the development of new drugs and for its extended use. Acute oral toxicity test is performed early on in the development stage to determine the effect of the single dose application for 1 or 2 weeks. This procedure also determines the median lethal dose (LD_50_) and provides a preliminary dose range [5]. A previous study on SHT reported its acute toxicity using Crl:CD Sprague Dawley (SD) rats and defined LD_50_ of SHT to be over 2000 mg/kg/day in rats, for both sexes [6].

To assess the no observed adverse effect level (NOAEL) and identify the most affected organ it is necessary to perform repeated-dose toxicity tests. These tests give information between continuous exposure of drug dose and animals, which is important for planning clinical trial [7]. To identify the relationship between the long-term exposure and the animal's response, we here assessed the 4-week repeated oral dose toxicity of SHT in SD rats. In this study, we investigated toxicity at higher dose (5000 mg/kg/day) than the one uses by Kim et al.'s study. This study aimed to build toxicity information, including NOAEL, and select a proper dose for subchronic toxicity test.

## 2. Materials and Methods

### 2.1. Preparations of SHT Water Extract

In order to obtain the powdered SHT decoction, the 9 raw materials, Paeoniae Radix (37.50 kg), Rehmanniae Radix Preparata (15.00 kg), Astragali Radix (15.00 kg), Angelicae Gigantis Radix (15.00 kg), Cnidii Rhizoma (15.00 kg), Zingiberis Rhizoma Recens (15.00 kg), Zizyphi Fructus (15.00 kg), Cinnamomi Cortex (11.25 kg), and Glycyrrhizae Radix et Rhizoma (11.25 kg), were purchased from Kwangmyungdag Medicinal Herbs (Ulsan, Korea), mixed, and extracted with 1,500 L of distilled water at 80°C for 2 h using reflux method. This preparation was conducted in Sungil Bioex Co. Ltd. (Hwaseong, Korea). The extracted water solution was spray-dried to make a powdered sample (39.0 kg, yield: 26.0%).

### 2.2. High-Performance Liquid Chromatography (HPLC) Analysis of SHT

Albiflorin (PubChem CID: 51346141, purity 99.8%), paeoniflorin (PubChem CID: 442534, purity 98.8%), ferulic acid (PubChem CID: 445858, purity 98.0%), liquiritin (PubChem CID: 503737, purity 99.6%), and glycyrrhizin (PubChem CID: 14982, purity 99.0%) were purchased from Wako Pure Chemical (Osaka, Japan). Cinnamic acid (PubChem CID: 444539, purity 99.0%), coumarin (PubChem CID: 323, purity 99.0%), and 5-(hydroxymethyl)-2-furaldehyde (5-HMF, PubChem CID: 237332, purity 99.0%) were purchased from Merck KGaA (Darmstadt, Germany). Nodakenin (PubChem CID: 73191, purity 99.5%) and liquiritin apioside (PubChem CID: 10076238, purity 98.0%) were purchased from ChemFaces Biochemical (Wuhan, China) and Shanghai Sunny Biotech (Shanghai, China), respectively. HPLC-grade methanol, acetonitrile, and water were purchased from J.T. Baker (Phillipsburg, NJ, USA). Glacial acetic acid for HPLC was analytical grade reagent, purchased from Merck KGaA (Darmstadt, Germany). The standard solution of each reference standard was prepared at a concentration of 1000.0 *μ*g/mL using methanol and was stored in the refrigerator. The HPLC analysis of extracted SHT sample was performed by modifying the previous study [8]. Briefly, HPLC system used was a Shimadzu Prominence LC-20A series (Kyoto, Japan) coupled with photo-diode array (PDA) detector. The ten marker components were separated using Gemini C_18_ (250 mm × 4.6 mm; 5 *μ*m, Phenomenex, Torrance, CA, USA) column maintained at 40°C. Mobile phases composed of distilled water (A) and acetonitrile (B), both with 1.0% (v/v) acetic acid, were flowed as follows: 5–60% B for 0–40 min, 60–100% B for 40–45 min, 100% B for 45–50 min, and 100–5% B for 50–55 min. The analysis was carried out at a flow rate of 1.0 mL/min and an injection volume of 10 *μ*L.

### 2.3. Animals and Maintenance

The animal maintenance was performed in the Korea Institute of Toxicology (earned AAALAC International accreditation in 2014). This study was conducted under the approval of the Institutional Animal Care and Use Committee (approval number: G217011) according to the “Guidelines for Toxicity Tests for Drugs and Related Materials, Document #2015-82” as prepared by the Korean Ministry of Food and Drug Safety.

Five-week-old Crl:CD SD rats (n=20/each of 10 female and 10 male) were obtained from Orient Bio Co. (Orient Bio Co., Ltd., Seongnam, Republic of Korea). All animals were housed under the controlled conditions of temperature (23 ± 3°C) and relative humidity (50 ± 10%) with a 12-h light/dark cycle, a light intensity of 150–300 Lux, and 10–20 air changes per hour. The animals were kept in stainless-steel cage for the observation period and allowed rodent diet (Catalog No. 5053, PMI Nutrition International LLC., MO, USA) and sterilized tap water* ad libitum*.

### 2.4. Group Assignment and Experimental Treatment

In a previous single oral dose toxicity test, SHT did not induce any toxic effect at dose up to 2000 mg/kg in Crl:CD rats [6] or 5000 mg/kg in CD-1 mice [9]. Considering the solubility of SHT and previous results, the maximum feasible dose was chosen at 5000 mg/kg/day. Based on the last body weight, healthy animals were randomly divided into four groups (n=5/group): SHT 1000, 2000, and 5000 mg/kg/day groups and a vehicle control group.

SHT was suspended in distilled water prior to each administration. The daily administration volume (10 mL/kg) was calculated based on the recent body weight of the individual animal. The vehicle control group had received distilled water.

### 2.5. General Observation

Mortality and clinical signs were monitored twice a day (before and after treatment) throughout the experimental period. The body weight and food intake were recorded once a week during the study period. Daily food consumption was calculated as the weight of the supplied diet and remaining in cages.

### 2.6. Clinical Pathology and Necropsy

Clinical pathology and necropsy method have been provided in a previous report [10]. Urine samples were collected at approximately 17 h in metabolic cages, and analyzed using Cobas U411 urine analyzer (Roche, Berlin, Germany), Combur 10 TM urine sticks (Roche), and TBA 120FR automated chemistry analyzer (Toshiba Co., Tokyo, Japan).

Animals were fasted over 17 h period prior to blood collection. Blood was taken from the postcaval vein under isoflurane anesthesia. Some of the blood samples were collected in a tube coated EDTA-2K. And then, we analyzed leukocyte count (WBC), total red blood cell (RBC), hemoglobin (HGB), hematocrit (HCT), mean corpuscular volume (MCV), mean corpuscular hemoglobin (MCH), mean corpuscular hemoglobin concentration (MCHC), relative reticulocyte (RET%), platelet count (PLT), and large unstained cells (LUC) using ADVIA2120i hematology analyzer (Siemens Healthcare, Erlangen, Germany). The rest of the blood samples were collected in a tube containing 3.2% sodium citrate. And then, we measured prothrombin time (PT) and activated partial thromboplastin time (APTT) using ACL Elite Pro coagulation analyzer (Instrumentation Laboratory, Milan, Italy).

For serum clinical chemistry parameters, collected blood samples were centrifuged at 3000 rpm for 10 min. And then, we analyzed blood urea nitrogen (BUN), creatine kinase (CK), albumin/globulin ratio (AG), aspartate aminotransferase (AST), alanine aminotransferase (ALT), gamma-glutamyl transpeptidase (GGT), total bilirubin (TB), total cholesterol (TCHO), triglyceride (TG), phospholipid (PL), and alkaline phosphatase (ALP) using a Toshiba 120 FR chemistry analyzer (Toshiba Co.).

After blood collection, the animals were euthanized by exsanguination from the postcaval vein and aorta. Absolute organs were measured and relative organs weights were calculated as a ratio of the following organs: brain, heart, lung, kidneys, liver, spleen, reproductive organs, thymus, thyroid and parathyroid glands, pituitary gland, adrenal gland, seminal vesicles with coagulating gland, and salivary glands.

### 2.7. Statistical Analysis

Data are expressed as mean ± standard deviation. The Bartlett's test was used to test the homogeneity of variances. When data had equal variance, group differences were assessed by a one-way analysis of variance (ANOVA) and a post hoc Dunnett's test using Pristima System (Version 7.2, Xybion, NJ, USA). When data variances were not equal, group differences were assessed by the Kruskal-Wallis test and a post hoc Dunn rank sum test.

## 3. Results

### 3.1. HPLC Analysis of SHT Decoction

**Figure 1:**
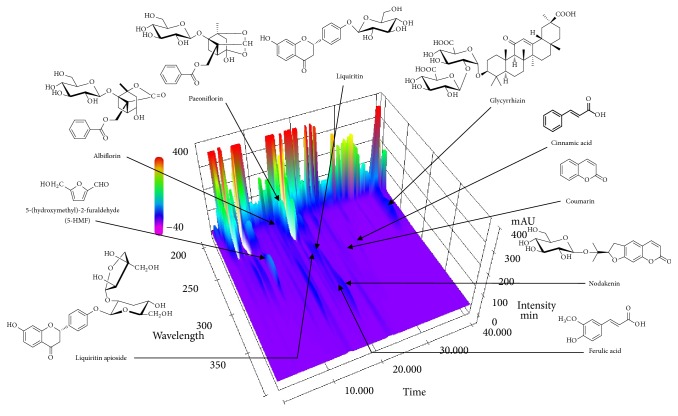
Three-dimensional HPLC chromatogram of the SHT samples using HPLC-PDA.

The established HPLC-PDA method was successfully applied to the quantitative analysis of the ten marker components in SHT. The stability of ten marker components in the extracted SHT was measured for 10 days (at 0, 1, 4, 7, and 10 days, respectively) using the prepared sample solution. As a result, the stability of all components was maintained at 95.9–105.5% as compared with the initial content at day 0 (Table 1). Ten marker components were eluted within 40 min and retention times of these components, 5-HMF, albiflorin, paeoniflorin, liquiritin apioside, liquiritin, ferulic acid, nodakenin, coumarin, cinnamic acid, and glycyrrhizin, were 7.85, 15.70, 16.62, 18.34, 18.76, 19.32, 19.76, 24.24, 27.74, and 38.50 min, respectively (Figure 1). The amounts of ten marker components in SHT decoction at 0, 1, and 4 weeks were in the range of 0.09–15.68, 0.09–15.63, and 0.09–15.81 mg/extract g (Table 2).

### 3.2. Mortality and Clinical Signs

No changes of mortality occurred in any of the groups during the experimental period (Table 3). Salivation was observed in male rats treated with SHT at 5000 mg/kg/day after 18-19 or 24 days treatment (Table 3).

### 3.3. Changes in Body Weight and Food Intake

**Figure 2:**
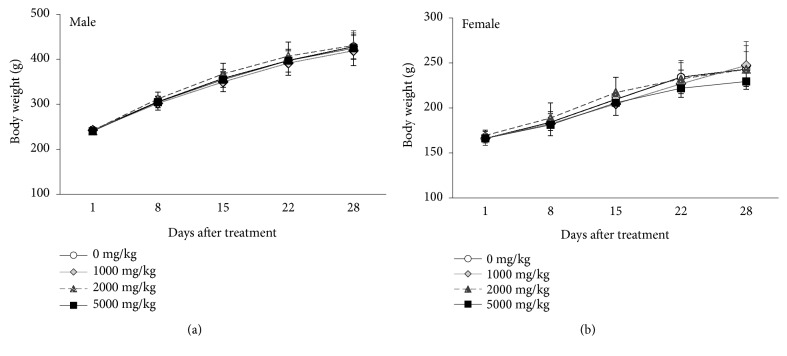
Body weight changes of male (a) and female (b) Crl:CD SD rats administered with the SHT at dose level of 0, 50, 100, and 2000 mg/kg/day for 4 weeks. Results are presented as mean ± SD.

In male rats, there was no difference in body weight changes upon treatment with SHT (Figure 2(a)). But, in female rats, the body weight tended to decrease at treatment at a dose of 5000 mg/kg/day of SHT after 22 days (Figure 2(b)).

**Figure 3:**
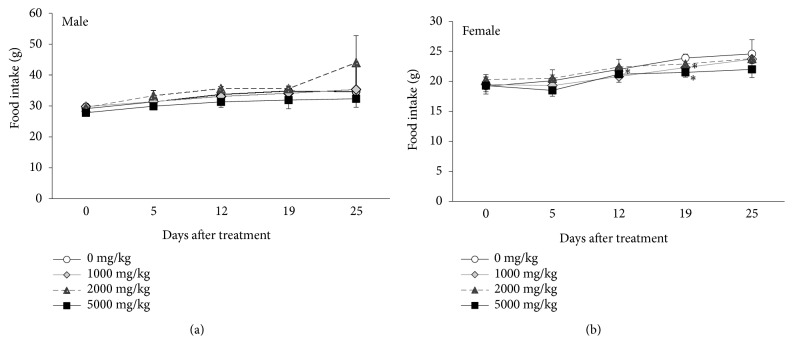
Food intakes of male (a) and female (b) Crl:CD SD rats administered with the SHT at dose level of 0, 50, 100, and 2000 mg/kg/day for 4 weeks. Results are presented as mean ± SD. *∗*Means significant at the* p*<0.05 level.

Food intake tended to decrease in male rats treated with SHT at a dose of 5000 mg/kg/day following 12 days of treatment (Figure 3(a)). In female rats, significant differences were observed following treatment with 5000 mg/kg/day (days 12, 19) and 1000 mg/kg/day (day 19) of SHT (Figure 3(b)).

### 3.4. Clinical Pathology

The level of RET% was increased in female rats treated with 5000 mg/kg/day of SHT (Table 4). The level of TB was increased in male and female rats treated with 5000 mg/kg/day of SHT. The level of ALT was decreased in male rats treated with 1000 mg/kg/day of SHT (Table 5). In urinalysis, the level of urine sodium was decreased in male rats treated with 5000 mg/kg/day of SHT. Urine bilirubin was observed in a male or female rat with 2000 mg/kg/day group, and in four male or five female rats in 5000 mg/kg/day group. Urobilinogen was observed in female rats in 2000 mg/kg/day group (n=1) and in 5000 mg/kg/day group (n=3) (Table 6).

### 3.5. Necropsy Findings

No treatment-related gross finding was observed in any rat. Relative liver weights were increased in rats of both sexes treated with 5000 mg/kg/day of SHT (Table 7). In female rats, relative weights of kidneys and spleen were increased in the group treated with 5000 mg/kg/day of SHT (Table 7).

## 4. Discussion

Herbal formulas, a complex mixture of herbs, have been widely used in the traditional clinic. They are prescribed based on the individual's constitution and taken for various days depending on symptoms. However, many of herbal formulas have not been subjected to toxicity tests. Drug toxicity is closely related to dosage and duration of exposure. Previous studies reported that SHT had no toxic effect at a dose up to 2000 mg/kg in Crl:CD rats [6] or 5000 mg/kg in CD-1 mice [9] in acute toxicity tests. In this study, we administrated SHT for 4 weeks in Crl:CD SD rats to evaluate subacute toxicity. The SHT treatment did not induce adverse changes in mortality, the body weight of male rats, and gross findings. Minor changes were observed in some of the rats treated with SHT.

The bitter taste is the most common flavor in the herbal formula, which induces secretion of saliva. Although salivation was observed in male rats treated with 5000 mg/kg/day of SHT, it was not accompanied with significant changes in salivary gland between the control group and the SHT-treated group at a dose of 5000 mg/kg/day. Therefore, we regarded the salivation, by administration of SHT, to be caused only by its bitter taste.

Oral administration of 5000 mg/kg/day of SHT significantly decreased the food intake in female rats at 15 and 22 days, which might also decrease the body weight. This finding did not show a dose-dependent correlation. Thus, any alteration in food intake and body weight in treated groups was regarded as incidental and not a sign of toxicity.

In 5000 mg/kg/day group, increased level of RET% in female rats, increased level of TB, and detection of urine bilirubin and urobilinogen might be considered as the SHT-mediated changes. A reticulocyte count evaluates bone marrow ability in erythropoiesis along with other tests including RBCs, HBG, and HCT. Although administration of SHT at a dose of 5000 mg/kg/day in female rats increased the RET%, it did not affect RBC level, nor the levels of HGB and HCT [11]. The normal or expected ranges for TB levels are as follows: 0.10-1.00 mg/dL for males and 0.20-1.00 mg/dL for females [12]. Although TB levels were significantly increased at the highest dose of SHT group compared to the control group, changes in TB level were within the normal ranges. Increased relative liver weight was observed in both sexes of rats treated with SHT at 5000 mg/kg/day; however, no changes were observed in gross findings and serum biochemical parameters of liver function such as AST, ALT, and GGT.

Measurement of the urinary electrolytes evaluates the renal function. Low urine sodium concentration was observed in the male rat treated with SHT at a dose of 5000 mg/kg/day. However, SHT did not change the serum electrolytes. Changes in kidney and spleen weight due to SHT were not observed in a dose-dependent manner and were just observed in single-sex of rats. Therefore, we considered these findings relatively of little toxicological importance.

## 5. Conclusions

Our findings demonstrated that SHT did not have any adverse effects in both sexes of rats up to a dose of 2000 mg/kg/day for 4-week administration period. Minor changes were observed at 5000 mg/kg/day (the highest dose tested), but SHT did not induce gross pathological findings. Further investigation, including subchronic toxicity study, is required to establish a firm conclusion. The same dosage, as used in this study, is considered appropriate to design a subchronic toxicity study.

## Figures and Tables

**Table 1:** Stability of ten marker compounds for 10 days in the SHT decoction (n=3).

Compound	Day (%)				
	0	1	4	7	10
5-HMF	100.0	102.2	104.0	101.4	98.9
Albiflorin	100.0	103.6	104.3	101.0	99.9
Paeoniflorin	100.0	98.1	99.7	96.1	95.9
Liquiritin apioside	100.0	100.3	99.7	101.1	98.1
Liquiritin	100.0	103.8	96.2	101.3	96.3
Ferulic acid	100.0	99.7	103.0	101.6	99.5
Nodakenin	100.0	100.7	100.4	101.4	100.2
Coumarin	100.0	99.3	97.9	101.5	99.0
Cinnamic acid	100.0	102.1	102.2	104.0	103.8
Glycyrrhizin	100.0	100.7	100.5	99.7	105.5

**Table 2:** Amounts of ten marker components in the SHT decoction at 0, 1, and 4 weeks by HPLC (n=3).

Compound	Amount (mg/extract g)									Source
	0 week			1 week			4 week			
	Mean	SD (×10^−1^)	RSD (%)	Mean	SD (×10^−1^)	RSD (%)	Mean	SD (×10^−1^)	RSD (%)	
5-HMF	0.94	0.06	0.66	0.92	0.18	2.00	0.92	0.19	2.08	RRP
Albiflorin	1.69	0.23	1.37	1.64	0.14	0.88	1.65	0.17	1.06	PR
Paeoniflorin	15.68	1.32	0.84	15.63	3.32	2.13	15.81	2.99	1.89	PR
Liquiritin apioside	3.51	0.41	1.18	3.41	0.56	1.64	3.52	0.52	1.48	GRR
Liquiritin	0.88	0.15	1.72	0.86	0.14	1.65	0.85	0.07	0.85	GRR
Ferulic acid	0.51	0.03	0.68	0.49	0.13	2.65	0.49	0.04	0.78	CR
Nodakenin	1.83	0.30	1.64	1.86	0.17	0.94	1.83	0.20	1.09	AGR
Coumarin	0.14	0.04	2.52	0.15	0.02	1.33	0.14	0.02	1.69	CC
Cinnamic acid	0.09	0.00	0.48	0.09	0.01	0.84	0.09	0.01	0.65	CC
Glycyrrhizin	7.00	0.56	0.81	7.00	0.84	1.20	6.95	1.99	2.86	GRR

RRP: Rehmanniae Radix Preparata; PR: Paeoniae Radix; GRR: Glycyrrhizae Radix et Rhizoma; CR: Cnidii Rhizoma; AGR: Angelicae Gigantis Radix; CC: Cinnamomi Cortex.

**Table 3:** Mortality and clinical signs in rats treated with SHT for 4 weeks.

Group	Dosing phase					Salivation^b^
	1 day	≤1 weeks	≤2 weeks	≤4 weeks	Final mortality^a^	
Male						
0 mg/kg/day	0	0	0	0	0/5	0/5
1000 mg/kg/day	0	0	0	0	0/5	0/5
2000 mg/kg/day	0	0	0	0	0/5	0/5
5000 mg/kg/day	0	0	0	0	0/5	5/5
Female						
0 mg/kg/day	0	0	0	0	0/5	0/5
1000 mg/kg/day	0	0	0	0	0/5	0/5
2000 mg/kg/day	0	0	0	0	0/5	0/5
5000 mg/kg/day	0	0	0	0	0/5	0/5

^a^Number of animals with dead animals/total animal number.

^b^Number of animals with sign animals/total animal number.

**Table 4:** Hematological parameters of rats treated with SHT for 4 weeks.

Dose (mg/kg/day)	0	1000	2000	5000
Male				

WBC (10^3^/*μ*L)	9.63 ± 1.77	7.83 ± 1.14	8.94 ± 1.13	9.56 ± 2.18
RBC (10^6^/*μ*L)	8.09 ± 0.26	7.88 ± 0.22	8.15 ± 0.20	7.82 ± 0.26
HGB (g/dL)	15.7 ± 0.47	15.5 ± 0.43	15.8 ± 0.64	15.3 ± 0.29
HCT (%)	47.9 ± 1.87	47.1 ± 1.34	48.5 ± 1.98	46.6 ± 0.66
MCV (fL)	59.3 ± 0.90	59.8 ± 1.66	59.5 ± 1.81	59.6 ± 1.50
MCH (pg)	19.4 ± 0.26	19.6 ± 0.53	19.4 ± 0.52	19.5 ± 0.67
MCHC (g/dL)	32.7 ± 0.44	32.8 ± 0.08	32.6 ± 0.53	32.8 ± 0.46
RET% (%)	2.52 ± 0.35	2.53 ± 0.19	2.37 ± 0.42	3.38 ± 0.95
PLT (10^3^/*μ*L)	1013.4 ± 53.30	1047.4 ± 88.88	908.0 ± 58.12	1027.8 ± 96.13
PT (sec)	14.8 ± 0.54	14.5 ± 0.45	15.2 ± 0.76	14.6 ± 0.61
APTT (sec)	16.1 ± 0.23	16.0 ± 0.92	16.3 ± 0.94	15.4 ± 0.47
Neutrophils (%)	13.3 ± 3.41	13.1 ± 3.55	12.2 ± 2.94	12.1 ± 4.87
Lymphocytes (%)	81.9 ± 4.63	82.4 ± 4.29	82.8 ± 3.70	82.9 ± 5.71
Eosinophils (%)	1.0 ± 0.23	1.0 ± 0.19	1.2 ± 0.50	0.9 ± 0.39
Monocytes (%)	2.3 ± 1.11	1.9 ± 0.41	2.2 ± 0.59	2.2 ± 0.70
Basophils (%)	0.3 ± 0.10	0.5 ± 0.29	0.3 ± 0.13	0.3 ± 0.05
LUC (%)	1.0 ± 0.18	1.2 ± 0.55	1.2 ± 0.19	1.6 ± 0.67

Female				

WBC (10^3^/*μ*L)	6.77 ± 1.121	7.00 ± 1.406	5.69 ± 1.119	6.46 ± 1.433
RBC (10^6^/*μ*L)	8.16 ± 0.498	8.17 ± 0.460	8.22 ± 0.335	7.62 ± 0.370
HGB (g/dL)	15.8 ± 0.52	15.9 ± 0.67	15.9 ± 0.58	14.8 ± 0.71
HCT (%)	48.2 ± 2.24	48.0 ± 1.59	48.5 ± 1.46	45.6 ± 2.02
MCV (fL)	59.1 ± 1.42	58.8 ± 1.75	59.1 ± 0.83	59.9 ± 1.89
MCH (pg)	19.5 ± 0.72	19.5 ± 0.61	19.4 ± 0.45	19.5 ± 0.76
MCHC (g/dL)	32.9 ± 0.96	33.2 ± 0.51	32.8 ± 0.67	32.5 ± 0.58
RET% (%)	2.10 ± 0.20	2.50 ± 0.85	2.71 ± 0.78	4.76^*∗*^ ± 1.40
PLT (10^3^/*μ*L)	1006.6 ± 125.33	1106.4 ± 102.83	991.2 ± 160.96	1012.2 ± 117.65
PT (sec)	14.7 ± 0.54	15.2 ± 0.41	15.1 ± 0.54	14.9 ± 0.59
APTT (sec)	13.9 ± 0.93	13.3 ± 1.83	13.6 ± 1.10	13.7 ± 1.58
Neutrophils (%)	12.3 ± 3.25	11.5 ± 3.9	10.8 ± 4.2	14.8 ± 6.2
Lymphocytes (%)	83.3 ± 2.16	84.2 ± 3.9	84.4 ± 4.1	81.2 ± 6.5
Eosinophils (%)	1.1 ± 0.54	1.0 ± 0.1	1.1 ± 0.5	0.9 ± 0.3
Monocytes (%)	1.6 ± 0.48	1.8 ± 0.3	1.8 ± 0.4	1.5 ± 0.3
Basophils (%)	0.5 ± 0.15	0.4 ± 0.1	0.4 ± 0.1	0.4 ± 0.1
LUC (%)	1.3 ± 0.43	1.1 ± 0.4	1.4 ± 0.4	1.2 ± 0.3

Results are presented as mean ± SD. ^*∗*^Means Dunn's rank sum test significant at the 0.05 level. WBC, total leukocyte count; RBC, total red blood cell; HGB, hemoglobin; HCT, hematocrit; MCV, mean corpuscular volume; MCH, mean corpuscular hemoglobin; MCHC, mean corpuscular hemoglobin concentration; RET%; relative reticulocyte, PLT; platelet count, PT; prothrombin time; APTT; activated partial thromboplastin time; LUC, Large unstained cells.

**Table 5:** Serum biochemical parameters of male rats treated with SHT for 4 weeks.

	Male				Female			
Dose (mg/kg/day)	0	1000	2000	5000	0	1000	2000	5000
Glucose (mg/dL)	106.8 ± 24.71	92.3 ± 21.73	98.0 ± 21.75	98.2 ± 17.86	91.3 ± 22.94	86.4 ± 29.49	93.6 ± 32.92	107.0 ± 34.19
BUN (mg/dL)	13.2 ± 1.25	15.3 ± 3.35	14.5 ± 3.18	14.7 ± 1.44	19.5 ± 3.05	15.7 ± 3.13	17.8 ± 4.16	17.6 ± 1.95
Creatinine (mg/dL)	0.48 ± 0.039	0.47 ± 0.050	0.51 ± 0.034	0.49 ± 0.067	0.53 ± 0.082	0.51 ± 0.078	0.54 ± 0.082	0.52 ± 0.065
Creatine kinase (IU/L)	666.2 ± 104.26	867.8 ± 245.02	887.2 ± 357.40	553.8 ± 244.37	608.8 ± 132.36	588.0 ± 198.72	647.4 ± 140.40	602.8 ± 143.98
Total protein (g/dL)	6.16 ± 0.25	5.96 ± 0.11	6.34 ± 0.37	6.14 ± 0.161	6.54 ± 0.35	6.27 ± 0.27	6.38 ± 0.17	6.55 ± 0.22
Albumin (g/dL)	4.08 ± 0.078	4.01 ± 0.068	4.16 ± 0.227	4.11 ± 0.054	4.32 ± 0.173	4.26 ± 0.166	4.35 ± 0.103	4.39 ± 0.184
Albumin/globulin ratio	1.98 ± 0.17	2.07 ± 0.08	1.91 ± 0.02	2.03 ± 0.137	1.96 ± 0.13	2.12 ± 0.12	2.15 ± 0.09	2.04 ± 0.10
AST (IU/L)	114.3 ± 16.46	120.7 ± 7.31	129.4 ± 31.51	105.6 ± 20.73	112.2 ± 7.14	108.4 ± 16.00	113.9 ± 12.84	113.9 ± 15.20
ALT (IU/L)	32.5 ± 2.07	27.3^*∗∗*^ ± 1.55	30.9 ± 6.25	29.0 ± 1.77	23.1 ± 3.40	24.7 ± 4.58	24.0 ± 0.69	22.8 ± 4.89
GGT (IU/L)	0.36 ± 0.096	0.36 ± 0.098	0.46 ± 0.212	0.47 ± 0.148	1.00 ± 0.322	0.75 ± 0.448	0.82 ± 0.192	1.02 ± 0.205
Total bilirubin (mg/dL)	0.090 ± 0.0126	0.087 ± 0.0140	0.115 ± 0.0296	0.143^++^ ± 0.0251	0.108 ± 0.0197	0.115 ± 0.0158	0.129 ± 0.0129	0.18^++^ ± 0.0182
Total cholesterol (mg/dL)	69.2 ± 8.35	61.0 ± 9.30	64.4 ± 6.11	70.8 ± 20.39	64.6 ± 6.15	66.8 ± 9.88	70.8 ± 15.94	65.8 ± 14.60
Triglycerides (mg/dL)	19.2 ± 5.36	18.6 ± 7.22	23.3 ± 5.01	25.3 ± 9.48	11.00 ± 2.49	7.40 ± 1.41	12.10 ± 7.78	10.70 ± 6.05
Phospholipid (mg/dL)	97.4 ± 8.41	86.4 ± 9.84	93.0 ± 2.45	100.6 ± 21.87	110.0 ± 11.00	107.0 ± 11.47	118.2 ± 21.09	110.8 ± 18.29
ALP (IU/L)	559.6 ± 132.03	502.4 ± 83.83	557.6 ± 103.62	559.5 ± 131.21	375.4 ± 82.20	408.6 ± 118.19	147.4 ± 120.99	370.9 ± 67.94
Calcium (mg/dL)	10.5 ± 0.429	10.5 ± 0.182	10.6 ± 0.502	10.6 ± 0.153	10.85 ± 0.31	10.58 ± 0.33	10.65 ± 0.42	10.83 ± 0.26
IP (mg/dL)	10.4 ± 1.081	11.3 ± 0.846	10.7 ± 1.304	11.2 ± 0.671	10.32 ± 0.31	11.08 ± 0.71	10.85 ± 0.57	10.94 ± 0.70
Potassium (mmol/L)	6.70 ± 1.298	8.80 ± 0.704	6.90 ± 1.667	7.90 ± 1.484	7.47 ± 0.12	7.60 ± 0.42	7.89 ± 0.60	7.70 ± 0.45
Sodium (mmol/L)	144.6 ± 1.82	143.8 ± 0.45	146.2 ± 1.64	145.0 ± 1.58	143.0 ± 0.71	145.0 ± 1.58	143.4 ± 0.89	144.6 ± 1.52
Chloride (mmol/L)	98.4 ± 1.67	99.4 ± 0.89	98.4 ± 1.82	98.8 ± 1.79	100.2 ± 1.48	100.2 ± 1.48	100.0 ± 1.58	100.2 ± 0.84

Results are presented as mean ± SD. ^*∗∗*^Means Dunn's rank sum test significant at the 0.01 level. ^++^Means Dunnett's LSD test significant at the 0.01 level. BUN, blood urea nitrogen; CK, creatine kinase; AG, albumin/globulin ratio; AST, aspartate aminotransferase; ALT, alanine aminotransferase; GGT, gamma glutamyl transpeptidase; TB, total bilirubin; TCHO, total cholesterol; TG, triglyceride; PL, phospholipid; ALP, alkaline phosphatase; IP, inorganic phosphorus.

**Table 6:** Urinalysis parameters of female rats treated with SHT for 4 weeks.

Dose (mg/kg/day)	0	1000	2000	5000
*Male*				
Volume (mL)	21.4 ± 5.81	14.8 ± 2.28	28.4 ± 8.65	20.2 ± 13.90
Specific gravity	1.010 ± 0.0035	1.013 ± 0.0027	1.011 ± 0.0042	1.011 ± 0.0022
pH	7.0 ± 0.00	6.7 ± 0.27	7.0 ± 0.00	6.7 ± 0.27
Potassium (mmol/L)	108.29 ± 26.694	149.69 ± 27.031	93.26 ± 29.780	95.96 ± 28.503
Chloride (mmol/L)	41.2 ± 9.42	42.0 ± 5.34	34.2 ± 11.30	31.6 ± 8.35
Sodium (mmol/L)	33.6 ± 16.01	38.2 ± 7.09	21.4 ± 14.33	10.6^+^ ± 4.62
Bilirubin^a^	0/5	0/5	1/5	4/5
Urobilinogen^a^	0/5	0/5	0/5	0/5

*Female*				
Volume (mL)	16.4 ± 3.85	18.6 ± 5.08	16.4 ± 7.92	10.4 ± 4.34
Specific gravity	1.015 ± 0.000	1.013 ± 0.0027	1.015 ± 0.0079	1.017 ± 0.0045
pH	6.6 ± 0.22	6.7 ± 0.45	6.3 ± 0.84	6.2 ± 0.27
Potassium (mmol/L)	123.89 ± 31.436	101.60 ± 34.955	106.01 ± 76.483	112.67 ± 36.457
Chloride (mmol/L)	59.0 ± 8.43	51.8 ± 21.42	57.6 ± 43.18	67.6 ± 21.61
Sodium (mmol/L)	50.2 ± 26.08	40.0 ± 16.69	37.4 ± 23.35	37.2 ± 13.81
Bilirubin^a^	0/5	0/5	1/5	5/5
Urobilinogen^a^	0/5	0/5	1/5	3/5

Results are presented as mean ± SD. ^a^Number of animals with sign animals/total animal number. ^+^means Dunnett's LSD test significant at the 0.05 level.

**Table 7:** Relative organ weight (%) of rats treated with SHT for 4 weeks.

Dose (mg/kg/day)	0	1000	2000	5000
*Male*				
Body weight	393.7 ± 24.60	394.2 ± 29.80	400.3 ± 31.07	393.5 ± 30.70
Brain	0.514 ± 0.0131	0.536 ± 0.0509	0.502 ± 0.0514	0.521 ± 0.0582
Heart	0.350 ± 0.0390	0.363 ± 0.0150	0.355 ± 0.0478	0.345 ± 0.0312
Lung	0.398 ± 0.0320	0.384 ± 0.0168	0.394 ± 0.0300	0.395 ± 0.0297
Kidneys	0.839 ± 0.0346	0.914 ± 0.0379	0.898 ± 0.0603	0.936 ± 0.0673
Liver	3.295 ± 0.1160	3.331 ± 0.2191	3.427 ± 0.2521	3.740^++^ ± 0.1483
spleen	0.192 ± 0.0371	0.207 ± 0.0251	0.189 ± 0.0277	0.238 ± 0.0566
Testes	0.860 ± 0.0642	0.798 ± 0.0494	0.823 ± 0.0751	0.860 ± 0.0670
Prostate	0.135 ± 0.0316	0.123 ± 0.0290	0.100 ± 0.0235	0.122 ± 0.0142
Epididymis	0.273 ± 0.0298	0.263 ± 0.0201	0.260 ± 0.0284	0.304 ± 0.0584
Thymus	0.127 ± 0.0132	0.114 ± 0.0215	0.158 ± 0.0316	0.147 ± 0.0152
THPA	0.005 ± 0.0006	0.006 ± 0.0010	0.006 ± 0.0010	0.006 ± 0.0015
PITG	0.003 ± 0.0002	0.003 ± 0.0002	0.003 ± 0.0003	0.003 ± 0.0002
ADRG	0.016 ± 0.0029	0.017 ± 0.0032	0.015 ± 0.0027	0.014 ± 0.0006
SVCG	0.347 ± 0.0472	0.369 ± 0.0542	0.338 ± 0.0415	0.365 ± 0.0836
SALG	0.182 ± 0.0297	0.191 ± 0.0137	0.179 ± 0.0115	0.184 ± 0.0226

*Female*				
Body weight	227.4 ± 14.60	227.1 ± 18.89	230.6 ± 17.99	217.6 ± 18.29
Brain	0.809 ± 0.0497	0.822 ± 0.0858	0.844 ± 0.0774	0.854 ± 0.0482
Heart	0.376 ± 0.0236	0.399 ± 0.0269	0.375 ± 0.0166	0.394 ± 0.0179
Lung	0.524 ± 0.0063	0.520 ± 0.0204	0.489 ± 0.0320	0.515 ± 0.0258
Kidneys	0.899 ± 0.0173	0.953 ± 0.0684	0.943 ± 0.0432	1.016^+^ ± 0.0595
Liver	3.534 ± 0.0908	3.584 ± 0.1903	3.698 ± 0.1469	3.914^+^ ± 0.1393
Spleen	0.236 ± 0.0322	0.244 ± 0.0228	0.237 ± 0.0211	0.299^+^ ± 0.0198
Ovaries	0.047 ± 0.0033	0.043 ± 0.0065	0.415 ± 0.0059	0.045 ± 0.0072
UTEC	0.255 ± 0.1209	0.210 ± 0.0159	0.243 ± 0.0596	0.248 ± 0.0500
Thymus	0.177 ± 0.0225	0.196 ± 0.0078	0.209 ± 0.0347	0.191 ± 0.0247
THPA	0.008 ± 0.0015	0.008 ± 0.0007	0.008 ± 0.0016	0.009 ± 0.0013
PITG	0.006 ± 0.0006	0.006 ± 0.0004	0.006 ± 0.0007	0.007 ± 0.0005
ADRG	0.034 ± 0.0054	0.031 ± 0.0034	0.033 ± 0.0065	0.035 ± 0.0049
SALG	0.206 ± 0.0156	0.200 ± 0.0149	0.194 ± 0.0121	0.202 ± 0.0225

Results are presented as mean ± SD. ^+^Means Dunnett's LSD test significant at the 0.05 level. ^++^Means Dunnett's LSD test significant at the 0.01 level. THPA, thyroid and parathyroid glands; PITG, pituitary gland; ADRG, adrenal glands; SVCG, seminal vesicles with coagulating gland; SALG, salivary glands; UTEC, Uterus/cervix.

## Data Availability

Due to organizational restrictions, the data and materials will not be available.
